# Prevention of the Unpleasant Taste of Balsam Copaiva

**Published:** 1860-05

**Authors:** 


*Prevention of the Unpleasant Taste of Balsam Copaiva.*—Dr. Landerer observes that by keeping the balsam undergoes some change, conferring upon it a taste which is very repulsive to the patient. While, too, some sorts of balsam are of light yellow color, possess a mild aromatic odor and taste, and swim when dropped in water, there are other kinds which are brown in color, much less agreeable to the smell, are of a sharp, irritating taste, and often sink when dropped in water. He is inclined to believe that these changes depend upon the formation of a resinous acid. At all events, the addition of magnesia or prepared oyster-shell to old, thick, brown balsam, very much diminishes the disagreeableness of its taste. The addition of syrup is a still further improvement; and if immediately after taking the balsam, a cup of well-sweetened coffee be drank, the disagreeable after-taste will scarcely be perceived.—*Buchner's Repertorium.*

